# History, Socioeconomic Problems and Environmental Impacts of Gold Mining in the Andean Region of Ecuador

**DOI:** 10.3390/ijerph19031190

**Published:** 2022-01-21

**Authors:** Carlos Mestanza-Ramón, Robinson Ordoñez-Alcivar, Carla Arguello-Guadalupe, Katherin Carrera-Silva, Giovanni D’Orio, Salvatore Straface

**Affiliations:** 1 Department of Environmental Engineering, University of Calabria, 87036 Rende, Italy; salvatore.straface@unical.it; 2 Research Group YASUNI-SDC, Escuela Superior Politécnica de Chimborazo, Sede Orellana, El Coca 220001, Ecuador; 3 Green Amazon, Research Center, Nueva Loja 210150, Ecuador; robinson.ordoniez@espoch.edu.ec; 4 Escuela Superior Politécnica de Chimborazo, Riobamba 060104, Ecuador; c_arguello@espoch.edu.ec (C.A.-G.); katherin.carrera@espoch.edu.ec (K.C.-S.); 5 Department of Economics, Statistics, and Finance, University of Calabria, 87036 Rende, Italy; giovanni.dorio@unical.it

**Keywords:** gold, heavy metals, socio-environmental impacts, political management, environmental management

## Abstract

Mining in Ecuadorian territory comprises three stages of Ecuadorian history: pre-Columbian, colonial, and republican times. In its beginnings, this activity did not have regulations or a legal foundation. The first Mining Law dates back to 1830, and it has been modified until the most recent update in 2009. The Andean region consists of 10 provinces, 9 of which have registered gold concessions, the most important of which are Loja, Azuay, and in recent years, Imbabura and Pichincha, which are the provinces with the highest number of reported concessions. The objective of this study focused on analyzing the historical and current situation of Artisanal and Small-scale Mining (ASGM) and the emergence of large-scale (industrial) mining. For the elaboration of this study, different methodological techniques were used, such as literature review, field interviews, and expert judgment validation. The main findings show that the provinces of Loja, Azuay, Imbabura, and Pichincha are the most conflictive areas in the region due to the impacts caused by mining activities. In socio-economic terms, there are conflicts between inhabitants in favor and against these activities and problems associated with human health. In environmental terms, the findings suggest historical contamination of water sources by heavy metals, which has altered the surrounding aquatic and terrestrial systems. Finally, the study concludes that implementing public policies should be promoted to balance socio-economic and environmental aspects in gold mining activities in the Andean region of Ecuador, strengthening the use of new technologies and education to raise awareness of the serious effects of mining activities.

## 1. Introduction

Mining worldwide is currently experiencing a production growth that has never been seen before. The global market for minerals and precious metals has soared, and the quantities extracted have multiplied exponentially [1,2]. Gold (Au), recognized as one of the most sought-after and demanded elements in international trade, is also the most exploited precious metal in world mining production [3,4,5]. In the case of South America, member countries, such as Colombia, Peru, Bolivia, Chile, Brazil, and Argentina, are notable for Au production and exports, a market in which Ecuador has become increasingly competitive during the last few years as part of a strategy to diversify its productive output [6,7].

In Ecuador, Au mining dates back long before oil extraction began, and for many years it has been a way of life and subsistence for thousands of families [8]. In fact, the development of this activity in the national territory includes three stages of Ecuadorian history [9,10,11,12,13]: pre-Columbian, colonial, and republican times. During this time, gold in our country has been extracted legally and illegally as well as in artisanal and small-scale mining, the latter thanks to the strengthening of its productive processes, new forms of business organization, and legal clarification since the 1990s [14]. This activity in the country is considered a strategic activity with an increasingly strong presence in the framework of the country’s productive matrix, not only because of the important economic and employment generation involved but also because of the relevant role it plays in attracting foreign direct investment [15], aspects which have changed over time thanks to the improvement of certain policies and the great geological wealth that the national territory possesses. On the other hand, it was not until 2007 that large-scale mining was considered a strategic activity for the economic and social development of the country [12,15]; since then, it has consolidated as a sector with an important projection for the dynamization of the Ecuadorian economy.

Ecuador is a new player on the large-scale mining scene in the Latin American regional context. This entry on the scene, produced during Rafael Correa’s government, is part of the so-called neo-extractivist policies [3]. Specifically, the Ecuadorian government directly links future mining exploitation with reducing poverty and the country’s development by applying the principles of responsible mining and increasing benefits from taxes and royalties for social programs [7]. However, the commitment to large-scale mining is strongly questioned in the Andean country by various social organizations, grassroots communities, and academics, who propose seeking alternatives to extractivism that are not based on the aggressive exploitation of nature [10].

As noted above, Au mining has been developed for several years in Ecuador [16,17], which is why it has been necessary to implement policies and laws to regulate the activity and efficiently manage the territory and the resources obtained from Au mining and other types of minerals [13,18,19,20]. Currently, within the regulatory framework that governs the country’s mining activity, the Mining Law of 2009 is a legal body supported by sovereign and environmental principles that established greater State participation concerning the benefits of the mining industry even over private companies [13,21]. This law also established a new institutional framework consisting mainly of the Sectoral Ministry, the Mining Regulation and Control Agency (ARCOM), the National Institute of Geological and Mining and Metallurgical Research (INIGEMM), the National Mining Company (ENAMI EP), and the municipalities in their respective areas of competence [12,13]; this is aimed at achieving effective and timely administration in the sector.

In 2018, the country registered an Au production of 11.5 tons, placing it 10th among countries producing this mineral at the South American level [21,22,23,24]. As for the international market, the main countries to which gold ore was exported during January–May 2020 were the United States, Switzerland, Italy, the United Arab Emirates, and India [21,23,24]. Mining in the national territory constitutes a promising source of economic income, with investments of up to around USD 3.8 billion by 2021 [25], important resources for strengthening the country’s social sector.

Despite the socio-economic benefits of Au mining development in the country, the environmental impacts generated by this activity are disadvantageously classified as a source of pollution, which depends on the extraction methods, scale of operation, location, and characteristics of the receiving environment [26,27]. Concerning health, the impacts of Au extraction are concentrated on the exposure of workers to toxic substances, such as mercury (Hg) and cyanide (CN), especially in the manual and rudimentary practices of artisanal and illegal mining [17,28]. Similarly, the inadequate disposal of mine tailings and the infiltration of toxic substances into the soil and bodies of water can result in health problems for people, plants, and animals near the mining sites, as well as those located downstream from the Au extraction sites [29].

Finally, while it is true that Au mining is becoming increasingly relevant in Ecuador’s economic model, it is necessary to point out the environmental repercussions of this extractive process in the country from its beginnings to the present. Given this, the present study proposes a hypothesis that ASGM activities and the emerging large-scale mining are generating an increase in environmental impacts, human health problems, and socio-economic conflicts. To respond to this hypothesis, the objective is to analyze the historical and current situation and challenges of Au mining in the Andean region of Ecuador from a different perspective, including political (laws), socio-economic impact (population displacement, loss of livelihoods, changes in population dynamics, cost of living, water scarcity, and health effects), and environmental (biotic and abiotic). The methodology used was based on literature review, interviews, field observation, and expert judgment.

## 2. Materials and Methods

### 2.1. Study Area

**Figure 1:**
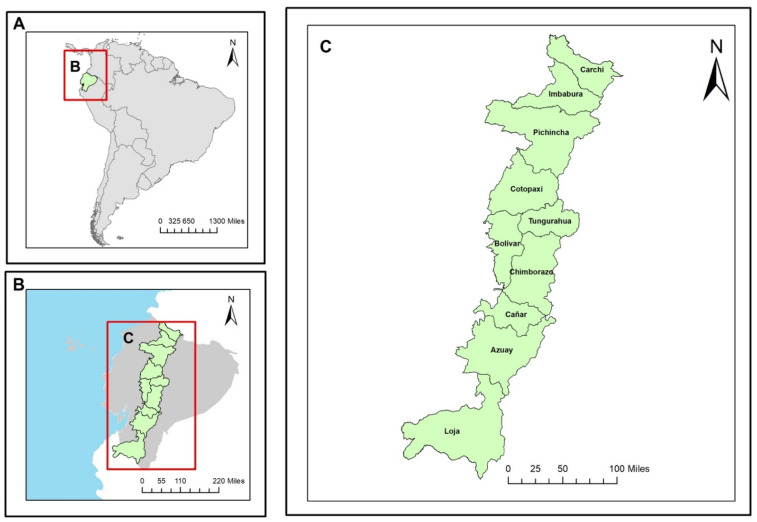
Study area: (**A**) geographical location of Ecuador; (**B**) Andean Region of Ecuador; (**C**) provinces belonging to the Andean region.

The Republic of Ecuador is located on the northwestern coast of the South American continent (Figure 1). The territory is crossed by the Equator Line, 22 km north of its capital (the city of Quito). It has a total surface area of 256,370 km^2^, distributed in two components: the continental area of 248,357 km^2^ and the insular area of 8010 km^2^ [21]. The continental zone, crossed by the Andes Mountains from north to south, is divided into three natural regions: the Coast or Littoral Region, the Highlands or Inter-Andean Region, and the Amazon or Eastern Region. In addition to these three regions, there is one more: the Galapagos Region in the insular zone.

Each of the regions into which the Ecuadorian territory is divided has different climatological and geographic characteristics, which have allowed it to have a great biodiversity per unit area [30]. Likewise, the characteristics of each region have allowed Ecuador to benefit from great geological wealth, finding mineral deposits that represent economic quantities of elements, such as gold, silver, copper, molybdenum, iron, titanium, etc. [31]; a fact that has aroused the interest of mining activity at all scales (artisanal, small, medium, and large scale) and in all its forms (legal and illegal).

This research focused on Au mining activity in one of the four regions of Ecuador, the Andean region, a region with extensive mountainous areas (Andes Mountains) made up of 10 provinces (Table 1). Au mining in each of these provinces was verified through the Mining Cadastre Web Geoportal [32] provided by the Mining Regulation and Control Agency (ARCOM), from which only those concessions registered in the aforementioned institution were filtered (Table 1).

### 2.2. Methods

The research was divided into three methodological sections. The first focused on a historical analysis and description of artisanal and industrial Au mining in the Andean zone of Ecuador. The second section examined the current situation of the same mining in the same study territory. Finally, according to the historical and current analysis, the third section discussed and pointed out the political, socio-economic, and environmental challenges involved in developing artisanal and industrial Au mining in the Ecuadorian Andean territory.

To answer the first and second methodological sections, it was necessary to conduct a systematic literature review focused on searching for and evaluating several papers published in journals and high-impact databases, such as Science Direct, Springer, Scielo, and Google Scholar. During the literature search and evaluation process, filters were applied by title, keywords, abstract, and year of publication (Table 2); this helped select recent documentation strongly related to the subject of study. From this selection process, eight documents on the historical context of Au mining in the Andean region and five on the current situation of artisanal and industrial Au mining in this same zone were obtained.

In addition to the literature review process (Table 2), the methodological process was complemented with an analysis of grey literature (Table 3) and information obtained from semi-structured interviews, field observations, documentaries, and web news (common search engines, social networks, and video recordings). The grey literature corresponds to a set of documents that have not undergone review, editing, or bibliographic control processes (ISBN, ISNN, Impact Indexes) and is available in non-conventional or low-coverage channels. The documents selected from this type of literature were laws, national plans, accountability reports, theses, and economic and research reports.

As for the semi-structured interviews and field observations, information was obtained from open-ended questions and informal conversations with key actors in the mining sector in the Andean zone of Ecuador (Table 4). Face-to-face interviews were preferred, with questions that allowed us to perceive the mining reality and to discover socio-economic, environmental, and political factors possibly not found in the literature. The interviews lasted from 30 to 60 min, and the interviewed audience consisted of affected people or inhabitants of the area, mining workers, and government authorities.

Finally, based on the bibliographic analysis in the first two sections and the information gathered in the interviews and field observations, a working group was formed. A method called expert judgment was applied, a process that allowed (i) validating the information obtained up to that moment, and (ii) discussing and establishing the main political, socio-economic, and environmental challenges surrounding the development of ASGM and large-scale mining in the Andean territory of Ecuador. The experts who participated were mining, environmental, and conservation issue specialists.

## 3. Results

Once the hypothesis was established regarding a possible increase in environmental impacts, human health problems, and socio-economic conflicts caused by ASGM activities that the emerging large-scale mining is generating, together with the methodological process set out in the previous section, it allowed us to respond to the objectives, which gave rise to the results presented below. For a better understanding, the political-legal aspects, socio-economic impacts (population displacement, loss of livelihoods, changes in population dynamics, cost of living, water scarcity, and health effects), and environmental (biotic and abiotic) aspects are described, with a historical and current perspective. Finally, challenges are set to contribute to the continuous improvement of ASGM and large-scale mining.

### 3.1. Evolution of Gold Mining in the Andean Zone

Mining in the national territory comprises three scenarios of Ecuadorian history: pre-Columbian, colonial, and republican times. The pre-Columbian era was characterized by the mining work of several pre-Hispanic cultures, including those who settled in the current provinces of Azuay and Cañar. The Cañaris worked Au with perfection, and an example of this is the mask of “El Sol de Oro”, found in 1940 in Chunucari, near the Sigsig canton [12]. Similarly, according to [12], Au mining during this period was dominated by the natives of the Inca Empire, with their offerings of *Kuri* (Gold) to the Sun God. This dominance was maintained until the colonial period, with the arrival of the Spaniards in America in 1492. It was during the colonial period that a milestone in the history of national Au mining was reached when the discovery of small Au particles in the effluent sands prompted the establishment of mining areas, such as Zaruma, Portovelo, and Nambija [12,33,34]. In the heat of the gold fever, the cities of Loja (1548, second foundation), Zamora (1549), Jaén (1549), Cuenca (1557), Valladolid (1557), and Sevilla de Oro (1575) were founded [12]. Nevertheless, the apogee of mineral extraction by the Spaniards lasted until the end of the 16th century, at which time the shortage of labor due to the decrease of aborigines and the reduction of shallow mines gave way to its decline [9,35].

However, in the republican era, mining in Ecuador took a new direction with the installation of the Great Zaruma Gold Mining Company, an ex-foreign company later sold to SADCO (South American Development Company) Portovelo y Zaruma—El Oro—Ecuador in 1897 [9,35,36]. SADCO undertook industrial Au mining and created the Cotopaxi Exploration Company, an entity established to exploit the Macuchi deposit in the Andean zone of the national territory (Cotopaxi province) [9,12,35]. Sometime after SADCO’s departure, in 1950, the Associated Industrial Mining Company (CIMA) was created and worked until the 1970s, leaving exploitation in the hands of small-scale miners and artisans [9,11,12,35,36]. In the 1980s, Nambija was rediscovered [33], and the Ponce Enriquez [34] and Cerro Pelado-Los Ingleses deposits were discovered and are currently being exploited [12]. In the 1990s, with the strengthening of artisanal and small-scale mining, new forms of industrial-type organization and changes in their legal framework were attracted. However, it was not until the government of the Citizen Revolution (2007 onwards) that mining activity, especially large-scale mining, came to be considered an option regarding changing the national production output, with the creation of strategic and second-generation projects [8,9,10,11,12,14].

Currently, ARCOM, through its Mining Cadastre Web Geoportal, contains about 797 registered Au mining concessions distributed in nine provinces of the Ecuadorian Andean territory. These concessions correspond to artisanal, small-, medium-, and large-scale mining, as well as several concessions belonging to the general regime group (Table 1). Likewise, in addition to the concessions registered in ARCOM, in the Andean zone of the country, there is evidence of illegal mining activity, which is clearly not registered but receives income without any oversight or control. Since the outset, illegal mining has represented a serious economic and social problem for the country due to the strategic income of which the State and the inhabitants of the mining areas are deprived [46].

Given this, according to [20], between January and October 2019, ARCOM, in coordination with the Mining Crimes Unit of the National Police and the Armed Forces, carried out 418 operations to combat illegal mining activities throughout the national territory. As a result, the number of offenders for this crime was reduced by 60% compared to 2018. However, by 2020 and to this day, a time in which the government’s attention has been focused on the COVID-19 health emergency and the country’s electoral process, illegal mining has not ceased. In fact, several complaints have been registered in the territory in protest of this activity, including those coming from Andean sectors, such as Buenos Aires (Imbabura), Pacto (Pichincha), La Rama (Loja), Ecuadorian Chocó (between Esmeraldas, Carchi, Imbabura, and Pichincha), Sigsig, and Ponce Enriquez (Azuay), among others. According to [38], thanks to this type of denunciation, between 2018 and October 2020, ARCOM has managed to seize 1733.27 tons of mining material in operations carried out nationwide, a figure that, depending on the Au content present in the material, would total between USD 15 and 20 million dollars for the State.

### 3.2. Political-Legal Aspect

Mining in its beginnings did not have regulations and a specific legal basis for its development, which generated rejection and misinformation in the population regarding this activity. The first Mining Law dates back to 1830, created during the government of Juan José Flores, to promote this activity. Subsequently, in 1937, a new law was issued that determined the State’s dominion over the minerals found in the subsoil, later codified by the Mining Law of 1961. Thirty years later, in 1991 and during the administration of Rodrigo Borja, an improved Mining Law was issued, which for the first time qualified this activity as one of national public utility and determined that mines and deposits are an inalienable and imprescriptible patrimony of the State. During that same year, the first General Regulation to the Mining Law was also issued, an instrument that designed the procedure for granting concessions and established tax and economic guidelines applicable to mining investment; this instrument was also modified in 2001 [12,14]. By 2008, with the issuance of a new Constitution that recognized nature as a subject of rights for the first time, the “Mining Mandate” was issued. This controversial document declared the extinction, without any economic compensation, of mining concessions in the exploration phase and had not submitted their environmental impact studies, generating a substantial pause in the development of the mining industry in the country [11,12,14,47].

Currently, the Mining Law and the Regulations governing the Ecuadorian territory since 2009, both issued with a series of guidelines that promote mining investment in the country and offer the opportunity to promote this industry within globally accepted parameters, require concessionaires to adopt mechanisms for environmental protection, employment generation, and development in the areas of influence. In addition, it allows the State to receive significant revenues through the payment of taxes, profits, royalties, and fees on extraordinary income, the latter being of strong relevance for the Ecuadorian economy [9,10,12,14]. On the other hand, since the year 2019, the Ecuadorian government has been planning an update to its comprehensive mining policy (Table 5) with the aim of improving mining management in the country and turning it into an activity that boosts the economy.

In interviews with 48 miners involved in ASGM activities in the different cantons in the Andean zone, 75% (36 miners) stated that they develop surface mining mainly in the mountains in the provinces of Imbabura, Pichincha, and Chimborazo. Conversely, 25% (12 miners) report that they mainly mine alluvial deposits in river beds and riverbanks, mainly in the cantons of the provinces of Azuay and Loja. Of the 48 miners, only 10, or 21%, indicated that they had permits or were in the process of regularization, mostly in the provinces of Imbabura, Azuay, and Loja. Of the 48 miners interviewed, only one representative of the Corazón mine in Cantón Intag, Imbabura province, stated that as an internal policy, 100% of their processes use cyanidation, and their wastewater is managed through a closed system. If it is necessary to discharge this water into bodies of water, it is first treated with hydrogen peroxide to reduce and eliminate cyanide in the tailings water, to comply with the environmental management plan submitted for the granting of the mining concession, as well as with Ecuadorian mining legislation and the regulations implementing the Minamata Convention on the non-use of mercury in mining activities [41]. Finally, in the rest of the provinces, miners stated that they are aware of the government policy prohibiting the use of Hg, but for economic reasons, practicality, and tradition, they continue with illegal amalgamation processes.

Regarding the creation of policies and laws, interviews were held with the environmental directors and their technical staff in the Autonomous Decentralized Municipal Governments. In all 48 jurisdictions, they agreed that policies aimed at developing control and monitoring of ASGM activities had not been discussed or analyzed. Thus, there was no evidence of ordinances adjusted to this end. The only evident thing is the personnel they provide to control operations carried out by the control authorities (ministry of the industry) and the public forces (police and military). On the other hand, 100% of the authorities state that they are aware of informal and illegal ASGM activities carried out in their jurisdictions, but it is difficult to carry out controls due to the high risk involved in moving to these isolated localities, considering that most miners are armed and could make an attempt on their lives. From the point of view of the provincial authorities in the 10 interviews held with the directors and technicians, they coincide with what was indicated by the cantonal authorities regarding the presence of informal and illegal ASGM activities in their provinces; they indicate that control is difficult due to the lack of personnel, low technical knowledge, and in the few controls that are carried out, there is evidence of information leakage, since, at the time of the operations, only evidence of ASGM activities is found, but not those responsible for them.

### 3.3. Socio-Economic Impacts

(a) Displacement of the population and loss of livelihoods

Regarding issues of population displacement, the results of the interviews indicate there are two opposing sides when analyzing ASGM and large-scale mining. On the one hand, ASGM is the option always present for the population of poor communities in the Andean zone. Historically, the vast majority of families in the Andean provinces have been economically dependent on agriculture and cattle ranching. Locals report that the population has been moving in recent years in search of better job opportunities to increase their income. One of the alternatives has been to engage in mining activities or be part of a new wave of migration to the United States and European countries since the borders were reopened after the period of confinement by COVID-19. Consequently, the traditional livelihoods on which they have historically depended, such as agriculture and cattle ranching, have been lost. This is reflected in the abandonment of agricultural and livestock lands, resulting in lower production.

Thus, unemployed or low-income people in the Andean Zone are happy to engage in ASGM activities. Of those interviewed in the cantons with mining activities, 100% indicated that at least one family member has participated in mining activities in the last five years. This has led to a decrease in local labor for activities, such as agriculture and livestock. In the seven cantons interviewed in the provinces of Carchi and Imbabura, locals are involved in ASGM activities in the La Merced de Buenos Aires region, a small parish in the canton of Urcuquí located high in the Andes Mountains in the province of Imbabura. Large-scale mining has led to the emergence of new rural settlements, as well as the displacement of indigenous peoples, who are generally evicted from their lands by large mining companies, both legally and illegally.

In Ecuador, illegal mining hotspots are mostly located in the parish of Buenos Aires (Imbabura) and in Camilo Ponce Enriquez (Azuay). Although only 0.38% of the national population is concentrated in these areas, 86% of the Au exported by small-scale mining is extracted there, and at least 50% of this comes from illegal and informal activities. However, despite such Au wealth, these sites have an average poverty rate of 65% for unsatisfied basic needs, according to data provided by INEC in its 2010 Population and Housing Census. Through informal conversations with some miners and local inhabitants, several agreed that they are part of the end piece of a millionaire system and perhaps the most precarious sector. In addition, several of them risk their lives in mines with a shortage of oxygen and the possibility of collapse; others tend to break into legal concessions to steal gold-bearing material and even request work in formal companies in order to physically swallow pieces of Au and silver, appropriate the precious metals in this way. Likewise, most miners agree on feeling gratitude for having a job that, although sometimes poorly compensated, is favorable to having none due to the health situation and the low economic level the country is currently going through. In addition, several miners pointed out that, at the time, they tried to legalize their activities through the corresponding agency, but due to the prevailing bureaucracy in the sector, the formalization process could not be completed. The formalization of activities opens up the opportunity to access credit and machinery needed in the sector.

Regarding large-scale mining, there has been anti-mining resistance in Ecuador, centered around different political positions and ethnic origins and arising in both urban and rural contexts. Despite the heterogeneous and interethnic nature of the anti-mining movement, the frictions between the government and the indigenous movement have led the latter, through the Confederation of Indigenous Nationalities (CONAIE) or the political party Pachacutik, to take up the mining issue as one of its main areas of protest and to lead the articulation of the opposition to the government. These tensions have been felt in relation to the discourse and rhetoric of the “indigenous philosophy” of Sumak Kawsay, due to the “appropriation” of the concept of Good Living by the Correa government, but also to the internal debates within the indigenous movement on the origins of the concept and its use as a political category.

(b) Population dynamics, economic growth, and cost of living

Historically, ASGM activities have been carried out mostly by men. In recent years, the participation of children, adolescents, and women in this activity has been increasing. Considering the different processes developed and their difficulty level, the different activities are assigned. The cost of living in the Andean zones has increased in response to a greater demand for products, which, over the years, are produced in smaller quantities, responding to a decrease in labor due to population displacement. It has also been evident that in the mining areas, the demand for basic products has increased, and there is a perception that the presence of miners has caused a rise in basic food prices.

For decades, in the socio-economic sphere, Au mining was the way of life and subsistence for thousands of native and immigrant families [8]. According to colonial records, in 1897, when SADCO began industrial Au mining in Portovelo, it produced more than 99 million grams until its dismantling in 1950, that is, approximately 1860 kg/year [9]. In 2000, the estimated small-scale production of gold ore by volume of extraction was 416.6 kg/month [14]. Between the years 2005 to 2012, the average Au production reached around 4900 kg/year, and for the period 2013 to 2016, an annual average of around 7700 kg/year was reached. However, a decrease in production was evident in 2016 and 2017, an aspect that would probably be explained due to the illegality and informality of ASGM, well as high levels of smuggling, though in 2018, it recovered, reaching a production of 6516 and reaching 8093 kg by 2020 [24].

Au mining and the extractive sector, in general, represent a productive activity that has served as a source of employment, income, and social investment projects generated by the State for thousands of people, either directly or indirectly, especially for the inhabitants of the communities near the place where these types of activities are concentrated. However, although a portion of the population has felt favored, there is another segment that considers itself encumbered and deprived of the benefits of the development of this extractive activity in the country. For example, several inhabitants living near the Au mines in Macuchi, an area discovered by the Cotopaxi Exploration Company and exploited since 1941, stated that, at that time, this area enjoyed a fairly good economic and social boom: “…it had perhaps the best hospital and theater in the country, sports fields, internationally renowned sportsmen…”. However, today this community in the province of Cotopaxi only shows the aftermath of looting, neglect, and contamination of its waters (Pilaló River) by arsenic and cyanide. In fact, there is a documentary made at the site entitled “Macuchi, half a Century of Contamination and Oblivion” [48].

In the interviews with the inhabitants of the populations living near ASGM activities, there are two points of view regarding quality of life. The first corresponds to families in which the heads of household depend directly or indirectly on ASGM activities. They state that, since their arrival, they have received higher incomes, and their quality of life has improved. On the other hand, families that do not depend on mining activities consider that ASGM activities have worsened their quality of life, such as environmental quality, water resources, and a decrease in local agricultural production. What these two parties agree on is that monthly income is generally not enough to cover basic expenses such as health, food, education, and clothing.

In the northern Andean zone in the province of Imbabura, in the La Merced sector of Buenos Aires, gigantic plastic camps were discovered that attracted attention. These camps contained beds and basic survival products and were used as a domestic refuge for illegal miners. Violence predominated in these sites, and those who lived there mentioned that it was an everyday occurrence, bringing fear and hopelessness. The local population that traditionally lived there clamored for help so that their territory would return to the tranquility that characterized it in the past. Several interviewed inhabitants claim that the Au rush attracted people from southern Ecuador as well as Peruvians, Venezuelans, and Colombians who intimidated the community, and even dissident guerrillas of the Revolutionary Armed Forces of Colombia (FARC) began to charge *vacunas*, economic payments in exchange for not attacking them, and to subdue the people in their attempt to dominate the illegal mining business.

At the beginning of July 2021, when the research team was conducting fieldwork in La Merced de Buenos Aires, one of the many control processes carried out by the government authorities was observed. On this occasion, approximately 1000 police officers, 1200 military personnel, and 20 prosecutors swept into the area in a lightning operation. In the first days of intervention, they managed to get around 3000 people to vacate the camps and to dismantle 30 Au processing plants and a complex system of pulleys for transporting Au. According to officials from the different ministries involved in the control and monitoring processes, it is estimated that some US 500,000 dollars a week were affected in Buenos Aires due to this illegal activity.

On the other hand, the arrival of large-scale mining in the Andean zone is causing important changes at the level of territorial control and use of natural resources, which generate a high level of socio-environmental conflict. In response to this phenomenon, the interethnic dimensions and the discourse of indigeneity emerged as very relevant elements in the articulation of local resistance to the Project [3]. While the indigenous inhabitants of the Andean zones consider themselves the legitimate owners of the territory and exercise their property rights through global titles, the mestizo peasants and ranchers are immersed in the process of protest against what they consider the irregular and unjust process of purchase of their individual titles, or the threat of being evicted from their lands under the figure of servitude [10]. Faced with this situation, the anti-mining resistance in the area has adopted the strategy of constituting themselves as indigenous communities and circumscribing their properties under a collective title, in addition to claiming the right to Free, Prior, and Informed Consultation, which is generally not carried out correctly.

(c) Water scarcity and health effects

A particular concern for the rural population is the impact of mining activities on water sources and streams that feed local aqueducts. Thus, the inhabitants of Andean mining areas associate water scarcity with four factors: (i) high demand for water from ASGM activities; (ii) increased demand generated by population growth in mining areas; (iii) lack of trust in the quality of water sources, which makes them unusable; and (iv) poor planning, which prevents dialogue-based solutions to the problem of water scarcity. Indeed, ASGM and large-scale Au mining activities are one of the main causes of water scarcity in addition to constant social demands due to economic and environmental problems. The use of toxic elements in the Au extraction processes and the lack of good environmental practices to reduce their impacts have caused serious damage to ecosystem components, mainly affecting water bodies, soil, and the atmosphere. As a result, there has been a decrease in the balance of ecosystems, which is reflected in the availability and use of its services. On the social side, water pollution contributes to increased health risks and decreased economic benefits.

Interviews with miners indicate that the most relevant health problems caused by mining activity are related to the nervous system, musculoskeletal system, respiratory, and psychological problems. This information was contrasted with data from the Ministry of Public Health showing an increase of 23% over the national average for respiratory ailments, intoxications, headaches, skin conditions, and vision problems. These problems are essentially caused by the release of heavy metals (mercury and zinc, among others) into water bodies, soil, and the atmosphere, in which they go through a chain of transformation to reach the human body. These problems are exacerbated by the lifestyles inherited from their ancestors, who lacked recreational activities, healthy physiological movements, and hours and days of real rest.

On the other hand, it is important to note that gold mining has caused occupational hazards due to the use of Hg and other heavy metals involved in the amalgamation [49] and cyanidation processes [50], as well as geographical incidents typical of this activity [51], such as telluric movements, landslides, and sinkholes, among others. Thus, in Ponce Enriquez, one of the most important Au and Andean mining areas in Ecuador, several deaths have been recorded due to geological accidents, poor technology (especially in illegal mining), and inadequate handling of certain materials [52,53,54]. Likewise, due to the irresponsibility and negligence present throughout the country’s mining history, which has led to serious environmental problems (discussed below in the third aspect of analysis), mining has become a highly controversial issue in Ecuador. For several years, various social movements have criticized the government in its attempt to promote mineral extraction in the country, arguing that mining, especially large-scale mining, is not compatible with the definition of social progress as “good living” [10,55].

In Ecuador, the risk of water contamination from large-scale gold mining activities is increasing due to the abundant and growing rainfall in recent years due to global climate change. Most of the current mining projects are located in highly rainy Andean areas (for example, it rains between 2500 mm and 3000 mm per year). Mining projects in paramo areas could cause an imbalance with serious consequences for water resources and surrounding life. Currently, 12.5% of the surface area of the paramo ecosystem is under mining concession [54]. In short, the problem of environmental impacts associated with large-scale mining stems from two main sources that can cause contamination over various periods and in different magnitudes. The first involves the extraction of huge quantities of rock from the subsoil that contain a wide variety of chemical elements: arsenic, lead, chromium, cadmium, sulfur, etc.; and once they reach the surface they are altered by rainwater or air, releasing these elements in dangerous quantities into the water, soil, and air. Second, when the rock is treated with toxic chemicals, these residues remain stored for years, and their impact on the natural environment is inevitable.

(d) Environmental Impacts

As a preamble, it is important to highlight the information provided by the ten provincial Environmental Directorates (director and technical team). According to the records, it was observed that between three to six complaints per month were received between 2015 and 2019 requesting intervention and monitoring of activities potentially causing socio-environmental impacts. As a result, the authorities organized a total of 143 inspections per year on average over the same period in order to respond to these requests in the Andean region.

A teleconference was held with representatives of the Environmental Quality area of the Ministry of Environment, Water, and Ecological Transition of the 10 provinces. As the environmental authority, they stated that environmental audits had been carried out on environmental licenses for ASGM activities, as indicated in Ministerial Agreement 061. As of 2020, 64% (452 mining concessions/rights) have submitted and approved environmental compliance reports, while 36% (250 mining concessions/rights) have not submitted environmental compliance reports. The provinces with the highest compliance are Imbabura and Loja, while Azuay and Carchi have the lowest percentage of compliance. In the audits in the territory, the environmental authority has not conducted environmental monitoring (biotic/abiotic) to compare with the results presented in the compliance reports due to a lack of budget, being tied to rely solely on the monitoring presented by the ASM concession representatives. In addition, in the audits carried out, the technicians state that they have found evidence of mining waste accumulation and leaching in 237 concessioned areas and sites adjacent to these activities, mainly in the provinces of Imbabura (2), Pichincha (15), Azuay (99), and Loja (121).

Furthermore, local miners state that Hg is used in ASGM gold-extraction processes, and some of it is lost when it is poured into the pans or processing ponds to form the amalgam. Another percentage of Hg is released into the atmosphere when the amalgam is burned. In the provinces of El Oro and Azuay in the south of the country, workers at the processing centers and miners who rent these centers on a daily basis claim they used Hg at some point in the last year. None of them knew about the final disposal of the water or simply indicated it was stored in pools. In other words, the water is not 100% subject to any treatment prior to discharge. Two very clear arguments emerged from the interviews with local miners involved in AGSM. First, artisanal miners who carry out alluvial and surface activities do not carry out procedures to mitigate the impacts generated in the different processes, arguing that they cannot cover the costs of applying procedures such as these. Meanwhile, in the processing centers, the workers or people who rent the centers do not know if, in the end, techniques are applied to mitigate the impacts of Hg and CN use. It is important to highlight that all interviewees agree ASGM has negative effects on water, soil, and air.

In addition to the information gathered in interviews with the main actors involved in ASGM activities, there was qualitative evidence of impacts, such as loss of vegetation cover, landslides, subsidence, soil erosion, water resources, and a decrease in the scenic beauty of the landscape in general. These impacts were recorded with greater magnitude in the provinces of Imbabura, Pichincha, and Azuay, generally generating pressures on biodiversity, affecting its balance and therefore causing a decrease in its ecosystem services. The following describes specific cases in mining areas where the impact on the natural environment is clearly evident.

**Figure 2:**
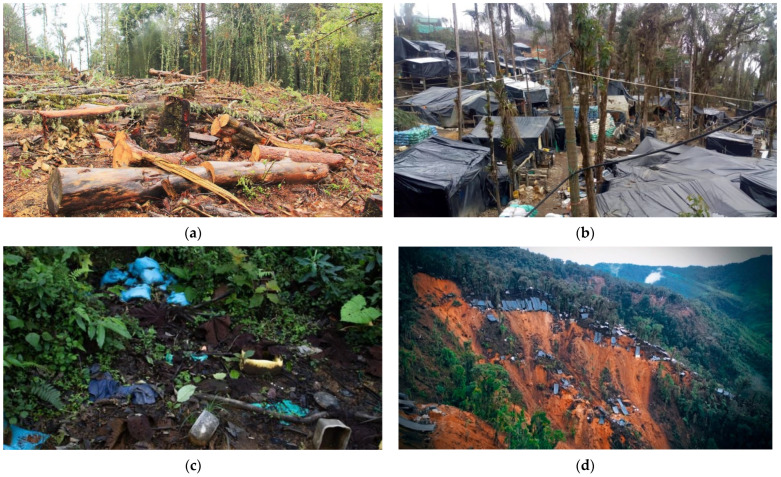
Environmental impacts; (**a**) vegetation clearing; (**b**) camp construction; (**c**) trash contamination; (**d**) erosion, landslides, and landslides.

In the mining area of La Merced de Buenos Aires, the mining boom generated since 2015 has had serious environmental consequences. The first impacts are caused when installing the precarious camps (Figure 2a) that will serve as shelter while mining activities are carried out. At this point, people cut down all the vegetation (Figure 2b). In addition, the presence of waste is evident (Figure 2c), which is not managed and mixes with the area’s biodiversity. This northern part of the country is characterized by mountains with high concentrations of Au in their rocks, which is why miners extract lumps of material, causing erosion and landslides (Figure 2d). At the same time, during the fieldwork, we located beneficiation plants that housed generators, fuel, tanks, and sacks of different chemicals (nitric acid, borax, sodium cyanide) in addition to ball mills, amalgamating cylinders, and cyanidation tanks, among others.

In the central Andean region, environmental impacts were also observed in the provinces of Pichincha and Cotopaxi. In the first province, located in the northwestern area, the environmental impact is of great concern to environmental organizations, considering that the area comprises legal concessions and illegal mining that directly affects the Andean Chocó Biosphere Reserve. This area is characterized by its high biodiversity and important water reserves for farmers, ranchers, and related communities. Meanwhile, in Cotopaxi, in the Macuchi Au mines, the locals say there is current contamination in their effluents due to the presence of arsenic and cyanide. A documentary made in the area indicates that during the mining boom that began in 1941 with the installation of the Cotopaxi Exploration Company, the area enjoyed a very favorable economic situation and social infrastructure: it had hospitals, shopping centers, movie theaters, large businesses, and economic mobility, among others [56]. Finally, in the southern zone of the Andean region in the province of Azuay in the Camilo Ponce Enriquez canton, environmental impacts continue to affect surface water bodies and groundwater.

Some environmental and social problems of large-scale mining activities are widely known. The development of this activity implies the generation of huge amounts of waste. The current trend in metal mining is the exploitation of low concentration deposits because the high concentration deposits are mostly depleted. To extract metal from low concentration deposits, mega-mining is necessary. However, this industrial activity involves the generation of waste in incalculable proportions. As far as Au extraction is concerned, waste levels are extremely high: the production of 10 g (equivalent to one ring) generates an average of 20 to 60 tons of rock waste, as well as 7000 L of water contaminated with cyanide, a substance known to be highly toxic [9,17]. The equation is simple: the lower the Au-content, the more waste. At the end of the mine’s useful life, millions of tons of tailings accumulate, with multiple risks of soil and water contamination [34]. The most common cases are acid mine drainage and heavy metals; air pollution is also frequently caused by dust laden with heavy metals, such as arsenic, cadmium, nickel, and even radioactive minerals [49].

Modern industrial mining involves the treatment of rock with highly toxic chemicals. The large amount of processed rock waste and water contaminated with these chemicals must be stored by miners for years. Although the industry claims that these wastes are safely controlled, contamination of the environment is inevitable. On many occasions, this contamination has proved catastrophic. The recent history of industrial mining recounts countless tailings dam ruptures caused by floods, earthquakes, or simple human negligence. In seismic regions or regions exposed to heavy rainfall, such as Ecuador, the risks of such dramatic accidents increase.

### 3.4. Challenges of Gold Mining in the Ecuadorian Andean Zone

Considering that deforestation, agribusiness, hydropower, and mining are the main threats that will continue to face the biodiverse ecosystems in the Ecuadorian Andes, the strengthening of environmental institutions should be one of the major challenges in Ecuador. Amid the COVID-19 pandemic, mineral and oil extraction activities continued as the main way to generate resources for Ecuador, so in 2020, in the face of a crisis and lack of employment in the population, Au mining increased. The governmental crisis forced ministries to merge in the environmental and control field, generating doubts about the strength that could be achieved to contain a growing Au mining.

Thus, one of the main challenges is to focus on improving the management and control of mining activity in the country and, above all, to discover whether the government sees this industry as an option for the economic reactivation of the country. In the political-legal sphere, it is necessary to establish more rigorous, clear, and realistic policies. According to [57], the categorical criteria for small and medium mining should be revised since they allow mining activity at much higher scales, with lax environmental management and low tax obligations. It is also important to focus on (i) insufficient environmental registration in artisanal mining to control its negative impacts, (ii) lack of coordination in mining-environmental-hydric permits, (iii) weak sanctioning processes, and (iv) a lack of technical guidelines in environmental management. On the other hand, in terms of control processes, miners call for attention to reduce bureaucracy and speed up the process of formalizing their activities. At the same time, inhabitants of affected areas request that ARCOM be more aware of this type of situation, as they claim that the agency only responds to complaints after media pressure.

In terms of the socio-economic aspect, the greatest challenge facing the country is the low level of transparency maintained by local and national authorities in the management of revenues. In view of this, the country must competently manage revenues obtained from mining extraction and exports and know how to invest them efficiently in the exploited regions, something that is being developed in part with the strategic projects of Loma Larga and Rio Blanco in Azuay, and with the royalties established by the national government. Another economic challenge facing the country is the rise or fall in mineral prices. This situation can cause financial instability but can be solved with the establishment of complementary and green economic activities in parallel to the extractive sectors, such as tourism, for example. The challenge in this area is for the Ecuadorian State to use the income obtained from the mining sector to invest in basic services and development in the exploited areas, while at the same time promoting economic activities that are less harmful to the environment and, in this way, become independent of the economic model based on the extraction of natural resources.

In recent years, governments have been betting on compensating for the programmed erosion of oil revenues with revenues from another extractive activity of the same kind: mega-mining. Indeed, like oil exploitation, mega-mining extracts non-renewable resources on a large scale and causes socio-environmental impacts of great spatial and temporal extension, perhaps even more important than those associated with oil extraction. The history of oil in the country shows a set of extremely high costs in social, cultural, environmental, political, and economic terms. It is from these that we must learn and not repeat the problems, considering that large-scale gold mining activity can cause catastrophic events if not properly managed.

Finally, concerning the environmental aspect, the Evaluation of Ecuador’s Mining Policy Framework [57] notes that, in terms of environmental management, the country lacks technical regulations, adequate control, and awareness of the environmental impacts of mining, specifically for proper management of (i) large volumes of solid and liquid waste, (ii) biodiversity in the exploration phase, (iii) water resources, and (iv) emergency preparedness programs and their socialization with communities and authorities. In addition to the above, inhabitants located in mining areas reported that there is a lack of continuous control and monitoring by environmental authorities to verify their activities, call attention to them, and prevent future incidents before they are already unavoidable; for example, the collapse of a tailings dam in Ponce Enriquez that resulted in the contamination of the Tenguel River [58].

## 4. Discussion

Historically and in recent times, the Andean Region in Ecuador has been characterized by increasing mining activity, especially in the southern zone, but this is not different from other regions such as the Littoral and Amazon [59,60]. Regarding social impacts, in the Andean Region they are mainly focused on human movement from the poorest villages to the mining areas, as this activity will always represent an opportunity to generate new income. These results are similar to those found in other countries, such as Colombia [61], Perú [62,63,64], Venezuela [65,66], and Bolivia [67], where migration problems present similar dynamics. In addition, it became evident that mining areas generally create new settlements and a greater demand for resources, increasing the cost of living at these sites. Socially, the natives are affected, and if they are not interested in joining the mining activity, they are forced to look for new locations involving less hostility. Similar situations have occurred in African and Asian countries [68,69,70,71].

Today, although the legal framework for mining has improved in recent years, the reality is that the country is still experiencing illegal mining and socio-environmental demonstrations against the government and its extractivist policies (several concessions have been granted in sites with high biodiversity, such as Quimsacocha, for example [36,41]), making mining at present a highly controversial issue. Illegal mining, as well as artisanal and small-scale mining, in many Latin American countries (such as Colombia, Peru, Bolivia, Chile, Brazil, and Argentina) is considered an activity with a marginal impact on the economy, depriving the State and the population of several investment and development benefits [2,32,35,49]. Similarly, this type of mining, characterized by the use of Hg and CN in its gold-extraction processes (a technique widely applied in countries such as Brazil, Colombia, Indonesia, Venezuela, and Zimbabwe), has meant that tailings contaminated with cyanide and heavy metals end up being discharged into water currents and the atmosphere [29,72], a fact that, due to poor management and technification, has caused the country several international problems. Such is the case of the Puyango-Tumbes river, shared with Peru, which was contaminated by traces of Hg coming from Portovelo-Zaruma [73,74]. The presence of illegal mining in the country, according to several informal workers, is because it represents their only source of income and that, in recent years, the country’s poor economic conditions and climatic phenomena have encouraged them to carry out this activity. They also point out that, on several occasions, they have tried to legalize their activities but that the bureaucratic system has prevented them from doing so [32,74].

## 5. Conclusions

Mining has become increasingly important as an engine of economic growth for Ecuador. The country’s great wealth of natural resources and the government’s policies of opening its economy to foreign capital have fulfilled their objectives of stimulating the mining sector. However, the benefits generated by the boost to this sector have been accompanied by numerous socio-environmental problems. The main causes of these problems have been associated with environmental impacts and territorial problems and linked in many cases to a lack of prior and informed consultation, the violation of human rights, a lack of Corporate Social Responsibility policies, and finally the struggle to obtain greater benefits by both the communities and the company and the local and central levels of government. It was also noted with some examples that governments have been reactively adapting mining legislation after problems have arisen. As a result, the economic, financial, environmental, and social costs have increased.

In the Andean region of Ecuador, Au mining has generated employment and local economic reactivation, mainly associated with lodging, food, and commerce by people involved in mining activities. However, these benefits have been minimal throughout history compared to the environmental impacts reported, such as the contamination of various effluents due to the presence of pollutants (e.g., arsenic and cyanide). In addition, there is evidence of damage to land use and forests due to the abandonment of areas exploited by illegal mining activities, where there are tools that were used, electrical plants, deteriorating housing, and a wide range of plastics, cables, glass bottles, clothing, and mattresses, among other things.

It is evident that large-scale mining causes environmental and socio-cultural impacts, which are very similar to those caused by other extractive activities. The profound modification of territories and economic activities caused by mega-projects implies a transformation of the human-nature relationship, destroys the material bases of indigenous peoples, brings new imaginaries of consumption and lifestyles, leads to a concentration of power, and therefore to an irreversible redefinition of the social structure of mining communities and their surroundings. In particular, it generates conflicts within the community and leads to the socio-economic marginalization of sectors unprepared for or traditionally considered less qualified for mining work and the related economic activities it generates, especially women and peasants.

In the study, the findings suggest that in the political-legal sphere, new policies should be established with a more rigorous approach and in accordance with the real needs of the population. In addition, the criteria for small- and large-scale mining should be re-examined, allowing activities to be carried out on a larger scale and under legal criteria. Therefore, it is of the utmost importance that the administrative processes to formalize these activities are quickly and efficiently initiated by the regulating entities. On the other hand, in the socio-economic aspect, it is evident that there is little transparency in the amounts obtained in the Au extraction and export process in the region, which should be invested adequately to mitigate the environmental impacts caused and boost the local economy of the area to change the traditional model used, which is based on the extraction of natural resources. In environmental terms, this study suggests that a key point would be to promote technical and legal regulations based on the adequate and effective control of illegal mining and encourage awareness programs regarding the environmental impacts associated with Au mining.

Finally, it is hoped that this document will be considered as a management tool and will serve to support authorities and social actors involved in Au mining in the Andean region in order to improve mining management processes. One of the main limitations of the study was the resistance in providing information for fear of reprisals by the power groups within the mining industry. Researchers are encouraged to carry out future complementary work on topics that allow a quantitative evaluation of the impacts on water bodies, soil, and atmosphere, and to associate them with the various socio-economic conflicts of the mining industry.

## Figures and Tables

**Table 1:** Registered Au mining concessions in the Andean zone of Ecuador.

No	Province	Canton	Concession Regime					Total
			Artisanal Mining	Small Mining	Medium Mining	Large Mining	General Regime	
1	Carchi	Tulcan	1	2	1	4	-	8
		Mira	-	-	-	6	-	6
		Espejo	-	-	1	4	-	5
	Subtotal		1	2	2	14	-	19
2	Imbabura	Cotacachi	-	3	2	11	-	16
		Ibarra	-	-	1	6	1	8
		San Miguel de Urcuqui	-	-	4	-	-	4
		Otavalo	-	-	-	1	-	1
	Subtotal		-	3	7	18	1	29
3	Pichincha	Quito	10	5	-	-	7	22
		San Miguel De Los Bancos	-	-	-	-	5	5
		Mejia	-	-	-	-	2	2
	Subtotal		10	5	-	-	14	29
4	Cotopaxi	Sigchos	1	-	-	2	-	3
		La Mana	6	9	-	2	-	17
		Pujili	-	1	-	-	-	1
		Pangua	-	3	-	1	-	4
	Subtotal		7	13	-	5	-	25
5	Tungurahua	--	-	-	-	-	-	0
6	Bolivar	Chillanes	16	2	-	-	5	23
		Guaranda	-	-	7	-	-	7
		Caluma	-	-	-	1	-	1
	Subtotal		16	2	7	1	5	31
7	Chimborazo	Alausi	2	-	-	-	-	2
		Riobamba	4	1	-	-	-	5
		Cumanda	10	1	-	-	-	11
	Subtotal		16	2	-	-	-	18
8	Cañar	Suscal	1	-	-	-	-	1
		Cañar	8	-	-	-	-	8
	Subtotal		9	-	-	-	-	9
9	Azuay	Cuenca	14	4	3	2	1	24
		Pucara	32	5	-	1	-	38
		Ponce Enriquez	57	42	-	3	5	107
		Santa Isabel	10	2	-	1	-	13
		Giron	-	-	-	1	-	1
		Sigsig	12	2	-	1	-	15
		Oña	-	-	1	-	-	1
		Paute	-	1	-	-	-	1
		Gualaceo	1	-	-	-	-	1
		El Pan	5	-	-	-	-	5
		Nabon	-	1	-	-	-	1
	Subtotal		131	57	4	9	6	207
10	Loja	Macara	139	15	-	-	-	154
		Celica	5	6	-	-	-	11
		Paltas	44	14	-	1	-	59
		Saraguro	4	1	-	-	-	5
		Loja	16	3	-	-	-	19
		Catamayo	12	2	-	-	-	14
		Zapotillo	14	1	-	-	-	15
		Olmedo	-	1	-	-	-	1
		Calvas	38	2	-	-	-	40
		Chaguarpamba	1	1	-	-	-	2
		Espindola	11	1	-	1	-	13
		Quilanga	23	-	-	-	-	23
		Sozoranga	57	-	-	-	-	57
		Gonzanama	14	-	-	-	-	14
		Puyango	3	-	-	-	-	3
	Subtotal		381	47	-	2	-	430
Total			571	131	20	49	26	797

**Table 2:** Selection process and literature review.

Topic	Keywords	Period	High-Impact Journals and Databases
Historical context of gold mining in the Andean region	“Gold mining in Ecuador” and “gold mining in the Ecuadorian Andean region”	Pre-Inca period–2018	[9,25,26,27,28,29,30,31]
Current situation of gold mining in the Andean Region		2019–2021	[32,33,34,35,36]

**Table 3:** Selected and reviewed grey literature.

Grey Literature	Registration	Citation
Foreign investment and mining policy in Ecuador	June 2017	[37,38]
Ecuador’s current mining legislation, including the Mining Code	1986	[39]
Mining Code Reform Law	1982	[40]
National Mining Sector Development Plan	July 2016	[41]
Organic Reformatory Law to the Mining Law, the Reformatory Law for Tax Equity in Ecuador, and the Organic Law of the Internal Tax Regime.	Official Gazette 037, 16-VII-2013	[42]
Integrated environmental management in the Puyango river basin	2013	[43]
Intervention in large-scale mining in Ecuador and violation of human rights	2010	[44]
Large-scale mining in Ecuador—Analysis and statistical data on industrial mining in Ecuador	2012	[45]

**Table 4:** Questions set to analyze the current situation of Au mining.

Participant	Questions
Local miner/Association representative (48 interviewees, mining concession owner)	What type of mining is developed? Do you have a permit to carry out mining activities? What type of technique is used for gold extraction, amalgamation, or cyanidation? Is wastewater in the extraction process subjected to some treatment process prior to its environmental discharge? Do you, as a miner, use any procedures to mitigate the impacts of gold mining? State three elements/components of the environment most affected by pollution. Over the years, do you think there has been a water shortage?
Local authority (48 interviewees, political leaders in the mining area)	In your jurisdiction, have ordinances been created to control and monitor mining activities? Do you know if there is illegal gold mining in your canton and/or parishes? Do you know if the inhabitants of your canton and/or parishes have had health problems associated with gold mining?
Ministry of Environment, Water and Ecological Transition (10 interviewees, government representatives per province)	Do you know if gold mining is developed in your province? Do you know if illegal gold mining is taking place in your province? Have there been reports of contamination from gold mining? How has the Environmental Authority developed audits of gold mining concessions (rights)? How has the Environmental Authority developed water monitoring in the water bodies in the mining influence zone? During the visits and/or audits, has the accumulation of mining waste been evidenced? Do you consider that leaching occurs in the residual accumulations?
Mining town residents (48 interviewees, community representatives per canton)	Do you consider that gold mining has improved the quality of life in the area? Does your monthly income cover all monthly expenses? Has gold mining caused population displacement for any reason? Has gold mining resulted in the loss of livelihoods? Has the cost of living changed since the advent of gold mining? Do you consider that gold mining activity causes a shortage of water for daily activities? Have any family members or acquaintances experienced health problems or death due to gold mining contamination?

**Table 5:** Mining public policy update.

Axis	Strategic Objectives	Public Policies
Economic development	Position the mining sector as a relevant industry in the national economy, promoting sustainable competitive investment.	To increase and diversify the production of the mining sector, encouraging national and foreign private investment in the long term.
Environmental and social sustainability	Promote the adoption of good environmental and occupational safety practices. Harmonize good relations between social actors in mining activities, providing for the development of the areas of influence through citizen participation.	Promote the responsible use of resources in a way that protects human and environmental health.
Research and development	Strengthen research by promoting technological development, technology transfer, and incentives for innovation.	Promote research, innovation, technology transfer, and entrepreneurship to develop the mining sector.
Management and administration	Articulate the functions and competencies of public institutions in the mining sector through intersectoral coordination.	Promote timely and efficient coordination in public administration.
Regulation, control, and fight against illegal mining	Strengthen regulation and control through auditing, supervision, and monitoring processes.	Improve the administration, regulation, auditing, and control of mining activities by the state.
Regulations	Promote a solid regulatory framework for the development of the mining industry by reviewing, analyzing, and proposing regulations that will lead to the sector’s safety.	Promote the updating and improvement of the mining regulatory framework to guide it towards sustainable development.

## Data Availability

Not applicable.
